# Graphene IoNanofluids, Thermal and Structural Characterization

**DOI:** 10.3390/nano9111549

**Published:** 2019-10-31

**Authors:** C. Hermida-Merino, A.B. Pereiro, J.M.M. Araújo, C. Gracia-Fernández, Javier P. Vallejo, Luis Lugo, M.M. Piñeiro

**Affiliations:** 1 Departamento de Física Aplicada, Facultade de Ciencias, Universidade de Vigo, E36310 Vigo, Spain; cahermida@uvigo.es (C.H.-M.); jvallejo@uvigo.es (J.P.V.); luis.lugo@uvigo.es (L.L.); 2 LAQV, REQUIMTE, Department of Chemistry, Faculdade de Ciências e Tecnologia, Universidade Nova de Lisboa, 2829-516 Caparica, Portugal; 3 TA Instruments Waters Chromatography, 28108 Alcobendas, Madrid, Spain; CGracia@tainstruments.com

**Keywords:** IoNanofluid, graphene, ionic liquid, rheology, thermophysical properties

## Abstract

Graphene is considered a promising substance in applications related to the capture and reduction of the environmental impact of fluorinated gases. However, further research is still required to explore all related possibilities. In this work, the potential use in this context of nanofluids (NFs), obtained by dispersing graphene nanosheets in fluorinated ionic liquids (FILs) is investigated. As a starting step, a thermal and structural characterization for this type of IoNanofluids (IoNFs) is presented. The highly nanostructured nature of FILs has been recently demonstrated. The presence of fluorinated moieties is responsible for enhancing the accommodation of solutes such as small gases. The strong tendency to self-assemble forming continuous and supramolecular structures, and the versatility to rearrange in several conformational features allows the stabilization of nano colloidal systems. It is essential to perform a comprehensive study of their structural features to understand the behavior of this type of heterogeneous systems. Therefore, we present screening on the phase and structural behavior of these novel IoNFs to discover and develop optimized systems where FILs turn out to be advantageous. Thermogravimetric analysis (TGA) was employed to evaluate IoNFs mass losses with temperature, and their solid–fluid phase transitions were located using a differential scanning calorimeter (DSC). Their rheological properties were also determined through oscillatory experiments, obtaining the viscous and loss moduli. In addition, the structural percolation transition was also identified.

## 1. Introduction

During the last decades, intensive research has been performed concerning ionic liquids (ILs) because they have been identified as environmentally friendly solvents in a wide range of applications [1], including chemical synthesis, purification processes, and catalysis [2,3,4]. Considering their structure, and comparing them with the traditional organic solvents, IL molecular structure is constituted by a large size and asymmetric organic cation and an anion of either inorganic or organic nature. The number of potential ILs is huge, due to the virtually uncountable combinations of different types of anions and cations, resulting in a formidable ability of dedicated fluid tailoring and design through the addition of different functional groups or variation in the cation alkyl-chain length [5]. ILs have very remarkable thermophysical, phase equilibria, and transport properties, and this includes virtually negligible vapor pressure, high thermal stability, tunable viscosity, and enhanced extraction capacity for diverse organic compounds and metals [6,7,8].

On the other hand, graphene nanosheets have been recently employed in the formulation of nanofluids (NFs). A survey of the literature reveals that this application of graphene in NF formulation is finding application, for instance, in heat transfer processes and energy production optimization [9,10,11], but as discussed in the present paper, there are several other potential applications. The target of this combination is to improve their heat transfer properties and tune their viscoelastic behavior. Graphene surface modification through chemical functionalization offers an innovative alternative to improve their stability in dispersion, which is usually reduced, as well as adjusting the thermophysical profiles and the transport properties of standard NFs. Moreover, the use of ionic liquids (ILs) as a base fluid is a successful route to further improve the stability and viscosity of standard NFs.

In particular, fluorinated ILs (FILs) are characterized by a higher molecular rigid structure and lower polarity as a result of the nature of carbon−fluorine bonds that promotes the formation of a nano segregated structure [12]. Their use is a promising option in extended applications range, such as flue gas extraction and separation, and capture of greenhouse gases. In this context, the combination with functionalized graphene nanosheets, included in the general category of IoNanofluids (IoNFs), allows new perspectives in this application. However, the structure and thermophysical profile of these new graphene IoNFs have not been characterized in detail so far.

In this work, we propose progress in the design of new absorbent media for the separation and capture of fluorinated gases (F-Gas). The atmospheric concentration of greenhouse gases (GHG) increases every year, contributing to the increases observed in the global temperature. In fact, most F gases have huge global warming potentials, some of them reaching 23,000 times the value of CO_2_, and their emissions have increased by 60% since 1990.

F-Gas are used as an alternative to chlorofluorocarbons (CFCs) and hydrochlorofluorocarbons (HCFCs) to prevent ozone depletion. In the literature, F-Gas have been shown to have high solubility in FILs, and thus, these innovative solvents have been proposed as separation and capture agents for these environmentally harmful gases. The combination of FILs with graphene nanosheets is proposed as a new step forward in this application. This tailoring of these innovative IoNFs is intended to enhance gas solubility through the selective adsorption of these gases on the surface of the nanosheets, which can be modified through chemical functionalization to increase affinity with F-Gas molecules. Nevertheless, the thermophysical and phase equilibrium transitions of these IoNFs are still poorly understood, and demand a rigorous characterization before considering them for the proposed application.

The use of IoNFs in this application presents some potential advantages if compared with traditional NFs. We can cite, for instance, the improved nanofluid stability due to the FIL viscoelastic nature, which reduces agglomeration and settling. In addition, and due to the IL negligible vapor pressure, no concentration drifts are produced due to the solvent evaporation during sonication, and this feature also improves the traditional NF performance.

With this objective in mind, the characterization of graphene-derived NFs will be performed using a highly surfactant FIL as a base fluid [13]. Preliminary studies have shown an extraordinary potential for this technical solution [14,15], but their development is still in the embryonic state due to the great complexity of chemistry and physics that govern the behavior of this type of system.

## 2. Materials and Methods

### 2.1. Materials

**Figure 1:**
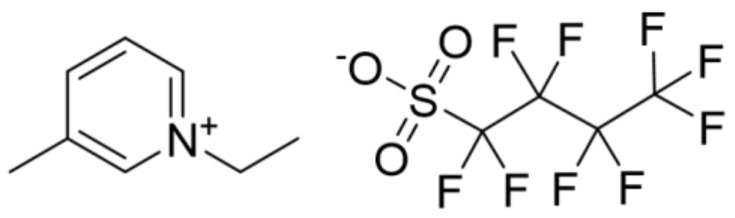
Structure of 1-ethyl-3-methylpyridinium perfluorobutanesulfonate ([C_2_C_1_py][C_4_F_9_SO_3_]).

The IoNF samples were obtained by dispersing exfoliated graphite nanosheets (xGnP) in a FIL. The xGnP supplier (XG Sciences, Inc., Lansing, MI, USA) provides structural information about the nanosheets’ geometry, with a surface area ≈750 mm^2^ g^−1^ and individual flake thickness in the range of 1 to 5 nm. Nano powder was weighed with a Mettler AE-240 balance, whose accuracy is estimated to be 5 × 10^–5^ g, and then dispersed into a calculated volume of the base fluid, obtaining stable and homogeneous xGnP/FIL IoNFs, with percent weight concentrations of 1, 5, and 10 wt%. An ultrasonic bath (Clifton, 80 W, Nickel-Electro Ltd., Oldmixon Crescent, UK) was used to obtain a correct dispersion. The FIL used in this case was 1-ethyl-3-methylpyridinium perfluorobutanesulfonate ([C_2_C_1_py][C_4_F_9_SO_3_]) (>99% mass fraction purity) supplied by Ionic Liquids Technologies. The thermophysical profile of this FIL has been determined in previous works [16,17], and its chemical structure is presented in Figure 1.

### 2.2. Methods

Thermogravimetric analysis (TGA) was performed through a simultaneous TGA/DSC (differential scanning calorimeter) device (Setsys Evolution 1750, Setaram, Caliure, France). With this technique, the thermal stability of the sample was evaluated, observing how the presence of graphene affects the thermal degradation of the ionic liquid. This allows selecting the temperature range in which the sample is stable, where all the following thermal tests were performed.

Isobaric heat capacities of the FIL base fluid, and of IoNF samples were experimentally obtained using a DSC Q2000 (TA Instruments, New Castle, DE, USA), with an estimated uncertainty of 3% [18,19]. This device was also used to determine the phase transitions of the xGnP/[C_2_C_1_py][C_4_F_9_SO_3_] IoNF. Cooling was carried out using a refrigeration and thermostatic system able to reach 183.15 K. Dry nitrogen gas was used to purge the sample, with a 50 mL·min^−1^ flow rate. An amount of 12 to 15 mg of each studied sample was sealed in a standard aluminum sample tray, resistant to pressures up to 0.4 MPa. Eventual mass losses during the measuring process were avoided by weighing the sample holder before and after the experimental runs. This verification is essential for experimental data validation. IoNF samples were kept under sonication for one hour to ensure correct dispersion before each experimental measurement. DSC calibration was performed using indium (melting point, *T*_m_ = 429.76 K) as standard. Samples temperature was then cooled to 183.15 K and maintained for 5 min. After that, they were heated to different temperatures, always respecting a minimum separation of 40 K between the last recorded transition and the cycle termination. Three repetitions of the cooling and heating cycles were performed for each sample at a constant 5 K·min^−1^ rate. This value of scanning rate ensures the best resolution and characterization of the various solid–fluid phase transitions. The DSC technique determines the thermal difference between two trays within a cell placed at the same conditions [20]. The first tray is filled with the sample studied while the reference one is empty. An endothermic or exothermic process occurring in the sample results, according to the “endo up” criteria, in a positive or negative peak in the thermogram recorded by the DSC. For the case of the Q2000 DSC, the heat flow between both trays can be calculated using [21]:

(1) $$\frac{dh}{dt}=\frac{\Delta T}{R_{r}}+\Delta T_{0}\left(\frac{1}{R_{s}}-\frac{1}{R_{r}}\right)+(C_{r}-C_{s})\frac{dT_{s}}{dt}-C_{r}\frac{d\Delta T}{dt}.$$

In this equation, *ΔT* is the temperature variation between the sample (*T_s_*) and the reference cell, *ΔT_0_* is the temperature difference between the sample and the sensor probe, *R_r_* is the sensor thermal resistance, and *C_r_* is the reference sensor heat capacity, and finally *R_s_* and *C_s_* stand for the sample sensor thermal resistance and heat capacity. These constants were calculated for temperatures in the range of 193.15 to 673.15 K. The uncertainties in both enthalpy and temperature were estimated to be 1.2 J·g^−1^ and 0.3 K, respectively.

The rheology study was obtained with a Physica MCR 101 Rheometer (Anton Paar, Graz, Austria). This device employs a cone–plate geometry (CP 50-1), and also a rugged plate–plate (PP50/S) 50 mm, with a constant gap of 0.048 mm. The torque range controlled by this rheometer is 0.5 to 125 mN·m. The experimental procedure employed to determine the rheological profile of different nanofluids has been detailed in previous works [22,23,24]. Linear viscoelastic measurements were carried out, and the determination of both store (G′) and loss (G″) moduli in the strain range between 0.1% to 1000% at an angular frequency fixed value of 10 rad/s, which led to the determination of the linear viscoelastic regime (LVR), at concentrations up to 10 wt% and constant temperature of 293.15 K. Frequency sweep measurements were then performed in the range between 0.01 and 100 rad/s, selecting a 0.1% strain value, in the same concentration and temperature conditions.

## 3. Results

### 3.1. TGA

**Figure 2:**
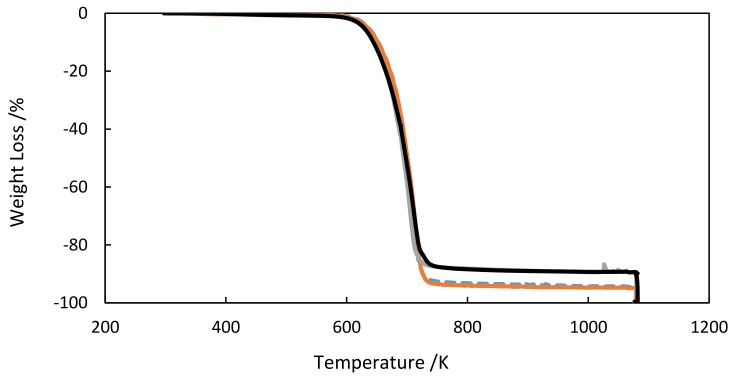
Thermogravimetric analysis (TGA) curves of xGnP/[C_2_C_1_py][C_4_F_9_SO_3_] at mass concentrations: (---) 0 wt%, (—)1 wt%, (—) 5 wt%, (—)10 wt%.

Thermal stabilities of the FIL base fluid and the xGnP/[C_2_C_1_py][C_4_F_9_SO_3_] were studied. A sample of 51 mg was heated from laboratory temperature to 1073 K at a constant rate of 5 K·min^−1^, maintaining an inert N_2_ atmosphere. Figure 2 illustrates the recorded TGA profiles. The results obtained are shown in Table 1. In the weight loss step, the sample remained constant until decomposition occurred above 600 K, with a weight loss below 6% and a degradation temperature *T*_onset_ = 686 K. Onset temperatures were obtained as the intersection between the baseline and the tangent to the derivative of the weight loss curve at the inflection point.

### 3.2. Phase Change Characterization

The solid–fluid transitions of the ionic liquid [C_2_C_1_py][C_4_F_9_SO_3_], used as the base fluid, and of the three IoNF concentrations (1, 5, 10 wt%) were analyzed by temperature scans with cooling and heating rates of 5 K·min^−1^. In addition, repeated cycling tests were performed with five repetitions for each sample at the same conditions, certifying in all tests the same transitions.

**Figure 3:**
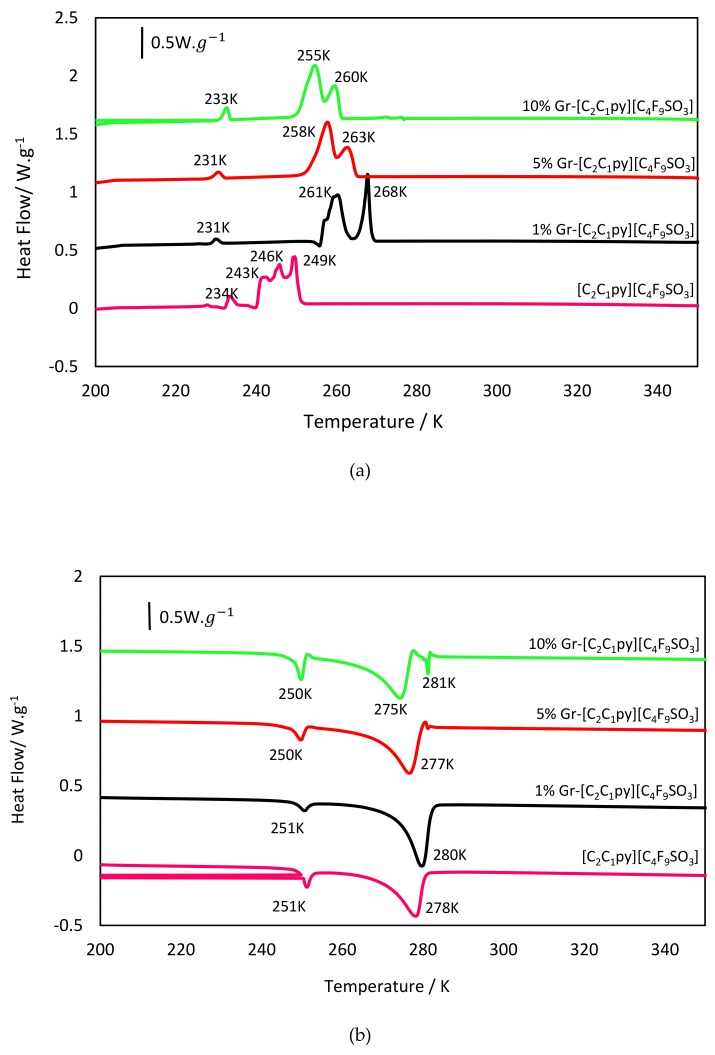
DSC thermograms of different concentrations of xGnP/[C_2_C_1_py][C_4_F_9_SO_3_]; at 5 K·min^−1^. (**a**) Cooling scan, (**b**) heating scan.

Figure 3 shows the cycles programmed for pure [C_2_C_1_py][C_4_F_9_SO_3_] and the xGnP IoNFs. The thermogram of the pure IL evidences that the material is polymorphic, presenting different solid phases in all cases. This behavior might be expected a priori because it has been shown [5,17] that FILs present a remarkably complex nano segregated fluid structure, with three types of nanometric size domains, namely, polar, nonpolar, and fluorinated. This structuration of the fluid phase leads to the formation of different types of solid crystalline structures, with a polymorphic behavior that is clearly evidenced by the thermograms.

If we focus on the cooling ramp, it is clear that the FIL, after the onset point at 251 K, presented six exothermic crystallization peaks, the first of them appearing at 250 K. Interestingly, after the third peak located at 243 K, an endothermic peak appeared before the next solidification. This indicates that the polymorphic solid presents enantiotropic behavior, which can be justified because there are different solid phases stable in different temperature ranges. This behavior means that one of the domains had a stable polymorphic phase in a certain temperature range, and the other crystal structure was stable in another temperature range. After this transformation, it recrystallized again, and that transition was clearly observed. Then, only two melting peaks appeared during the heating process, and the transition between the different solid phases could be resolved adequately.

The dispersion of graphene nanosheets, with a 1 wt% concentration, clearly changed the fluid–solid transitions of the FIL. The first effect was that the onset point rose eighteen degrees because graphene acts as a nucleating surface, promoting an earlier crystallization. The cooling ramp shows now three clear crystallization peaks. The second of them was followed by an endothermic peak, again evidencing an enantiotropic polymorphic transition. If compared with the pure FIL plot, some of the previous transitions did not appear in this case. This can be attributed to the presence of graphene nanosheets that on the one hand induce nucleation, but on the other hand, act as impurities hampering some of the previous solid–solid observed transitions.

The next essay was performed for a 5 wt% xGnP concentration. In this case, this concentration value was higher than the percolation concentration (that was calculated later in this work and will be shown to be located at 1.52 wt%). This means that the graphene nanosheets formed a 3D percolated structure, where the IoNF accommodated in the interstices and thus, decreased its mobility. This structural arrangement inhibited the enantiotropic solid domain transition, showing a more simplified solid phase transition scenario. Nevertheless, a new and interesting feature appeared in the heating ramp, as after the final fusion peak, appearing at 277 K, a clear exothermic peak is now displayed. This means that after the FIL had lost all its structure, a final solidification upon heating occurred, and this can be attributed to a transition of the percolated graphene network that produces a structured phase.

This last effect appeared even with more clarity in the 10 wt% concentration thermogram. In this case, the cited exothermic peak appeared at the end of the heating ramp, but also, an endothermic peak was clearly resolved around 277 K in the cooling ramp. This means that, before the FIL presented the already described liquid–solid transitions, a previous fusion of the remaining structural order of the percolated graphene was produced.

These results show, as the first conclusion, the very complex behavior of the perfluorinated IoNF solid–fluid transitions by itself. Then, this trend was remarkably modified upon the addition of graphene nanosheets. The final phase transition scenario combined features of enantiotropic and polymorphic solid–solid transition for the pure IL, the contributions of nucleation enhancement, solid transition frustration, and ultimate percolated structure of the dispersed graphene nanosheets.

### 3.3. Isobaric Heat Capacity

**Figure 4:**
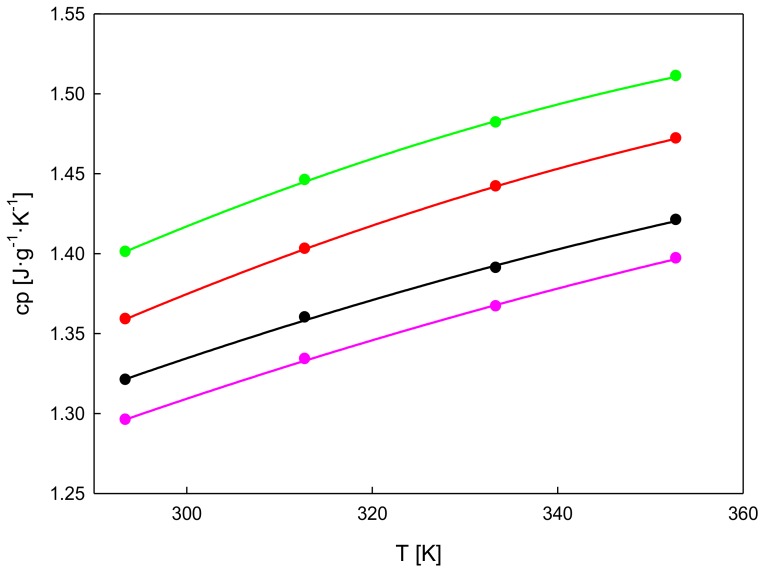
Isobaric specific heat capacity, *c_p_*, vs. temperature, of the xGnP/[C_2_C_1_py][C_4_F_9_SO_3_] at mass concentrations: (—) 0 wt%, (—)1 wt%, (—) 5 wt%, (—)10 wt% at 293.15 K.

The isobaric specific heat capacity, *c_p_*, was also determined experimentally. Table 2 lists the measured values for both the pure FIL used as the base fluid and also the different IoNF concentrations. Figure 4 presents the temperature dependence of isobaric specific heat capacity values, *c_p_*. The measured *c_p_* values increased with graphene concentration and temperature, with the highest value analyzed at the concentration of 10 wt% xGnP.

The literature about isobaric heat capacity for nanofluids shows different effects on the heat capacity of the base fluid by the dispersion of nano additives. Thus, according to the review by Riazi et al. [25], when the base fluid is water, a glycol or a mixture among them, the *c_p_* of the corresponding nanofluids tends to decrease with the nano additive loading. On the contrary, the majority of works with nanofluids based on molten salts showed *c_p_* increases derived from nanoparticle addition. In the case of ILs as base fluids, the literature shows more discrepancies depending on different parameters as the type of nano additive or the stability of the dispersion, among others.

An increment of heat capacity can be attributed to the formation of a solid-like nanolayer on the nanoparticle surface. This nanolayer contributes to the increase in specific heat of the studied IoNF.

### 3.4. Degree of Subcooling

**Figure 5:**
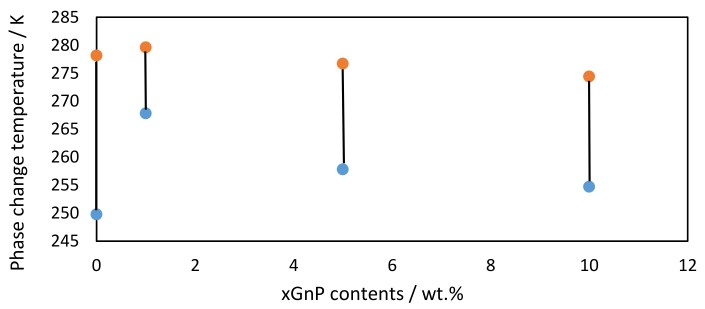
Phase change temperatures of pure IL and the prepared xGnP/[C_2_C_1_py][C_4_F_9_SO_3_] at different mass concentrations where: melting point (circle —); and freezing point (circle —).

The degree of supercooling of xGnP/[C_2_C_1_py][C_4_F_9_SO_3_] was also calculated due to its importance in different practical applications. This degree of supercooling can be computed from DSC results (listed in Table 3)**,** as the difference between the melting and solidification temperatures [26,27], and the numerical values are gathered in Figure 5, and compared with the pure FIL. The extent of supercooling in the case of xGnP/[C_2_C_1_py][C_4_F_9_SO_3_] was reduced by up to 58%. This result indicates that the extent of supercooling of the original FIL can be favorably reduced through the addition of graphene nanosheets. These nanosheets act then as nucleation promoters, resulting in that the IoNF exhibited improved nucleation and crystallization behavior if compared with the pure FIL.

### 3.5. Viscoelastic Measurements

#### Oscillatory Rheology

During the linear viscoelastic measurements, the linear viscoelastic regime (LVR) was determined as the first step by measuring both store (G′) and loss (G″) moduli of IoNFs in the strain range between 0.01% to 1000% at 10 rad·s^−1^ frequency, and 293.15 K, at concentrations up to 10 wt%. In addition, frequency sweep measurements were carried out from 0.1 to 100 rad·s^−1^ with a fixed strain value of 0.1%, for the same concentrations and temperature.

(1) Strain Sweep

In these tests, the influence of the deformation of the fluids at a constant frequency on the viscosity was recorded, determining the region of linear viscoelasticity (LVR).

**Figure 6:**
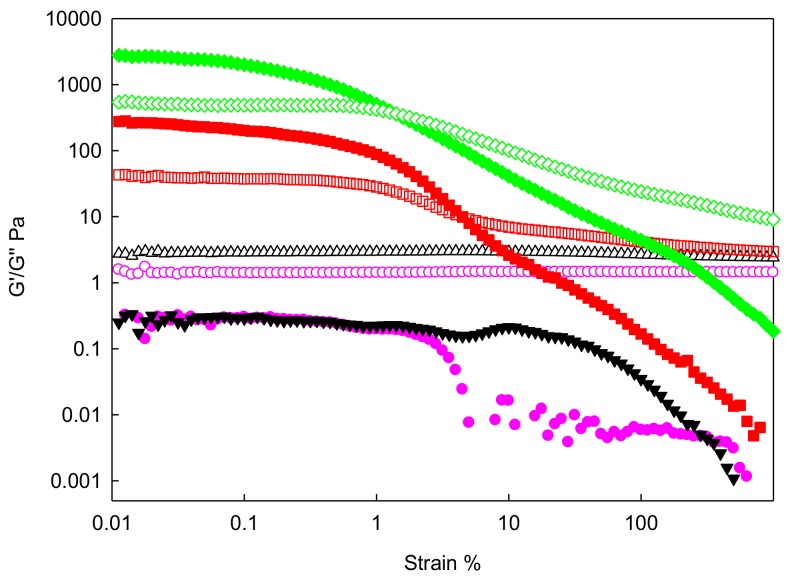
Store (G′, solid symbols) and loss (G″, empty symbols) moduli versus strain for xGnP/[C_2_C_1_py][C_4_F_9_SO_3_] at mass concentrations: (circle** —**) 0 wt%; (triangle **—**) 1 wt%; (square** —**) 5 wt%; and (diamond** —**) 10 wt% at 293.15 K.

The values of storage (G′) and loss (G″) moduli, plotted against strain variation, are shown in Figure 6. An amplitude sweep was determined for the FIL and different concentrations of graphene at 293.15 K. All of them have been carried out at 10 rad·s^−1^ constant frequency, with a deformation range from 0.1% to 1000% and twenty points per decade.

As can be seen, the elastic component reached a point where torque was so low that the equipment found its measuring limit.

It can be observed that the addition of nanoparticles produced an increase in both the elastic and viscous moduli because the dispersion had a higher resistance to flow. There was also a greater coherence in the data due to the arrangement in the structure of the ionic liquid with the aggregation of the nanoparticles.

The linear regime in the viscoelastic behavior is clearly observed, where G″ was constant and strain independent. Afterward, for higher deformations, both moduli decreased for all samples. The structure resisted up to a certain tension when the internal structure was lost by the disaggregation of the suspended nanosheets.

For the pure FIL and 1 wt% IoNF, the viscous modulus was larger than the elastic one. However, at concentrations above the percolation concentration (described below), the elastic modulus G′ was higher than the viscous modulus G″. Then, the sample presented an interesting transition in its viscoelastic nature, evolving from a liquid-like a behavior at low concentrations, to a solid-like trend at higher graphene loads. Once the region of linear viscoelasticity was determined, the frequency tests were carried out with a selected deformation value of 0.1%.

(2) Frequency Sweep

**Figure 7:**
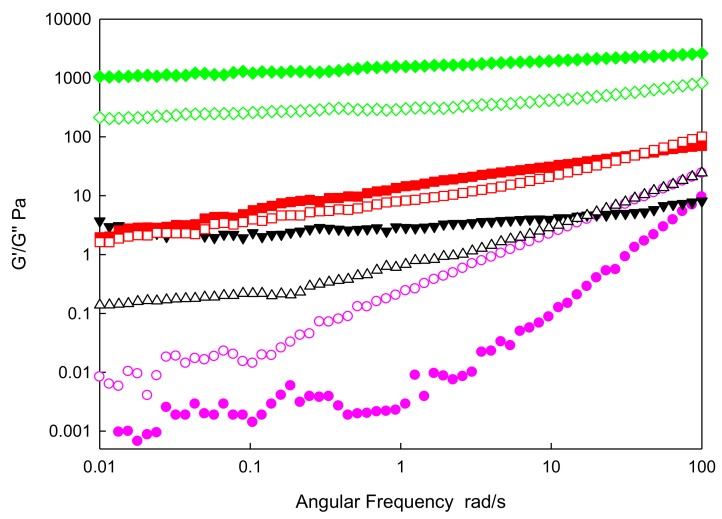
Store (G′, solid symbols) and loss (G″, empty symbols) moduli versus angular frequency at 293.15K for: xGnP/[C_2_C_1_py][C_4_F_9_SO_3_] at mass concentrations: (circle** —**) 0 wt%; (triangle **—**) 1 wt%; (square** —**) 5 wt%; and (diamond** —**) 10 wt%.

Figure 7 shows the data obtained for the base fluid and three IoNFs with a concentration of nanoparticles of 1, 5, and 10 wt%, in a frequency range from 0.01 to 100 rad·s^−1^ at 293.15 K.

As in strain sweep tests, the viscous component predominated over the elastic one in the entire region of linear viscoelasticity for the pure FIL, and it was the same case for low concentration of graphene. On the contrary, for concentrations above the percolation threshold, the opposite trend was observed. Then, the elastic modulus was predominant, being a feature of complex fluids approaching a solid-like trend. Both moduli increased with angular frequency, and also with the concentration of nanoparticles. This may be due to the structure of the pure liquid being unstable.

(3) Graphene Percolation

The determination of the concentration of graphene percolation was obtained by extrapolation. Thus, five concentrations of graphene IoNF (0, 1, 5, 7.5, 10, and 15 wt%) were analyzed. Then, it was necessary to obtain the plateau of elastic modulus, from a frequency sweep within the (LVR), at the same conditions than frequency sweep, and 293.15 K, to ensure that all material was melted.

**Figure 8:**
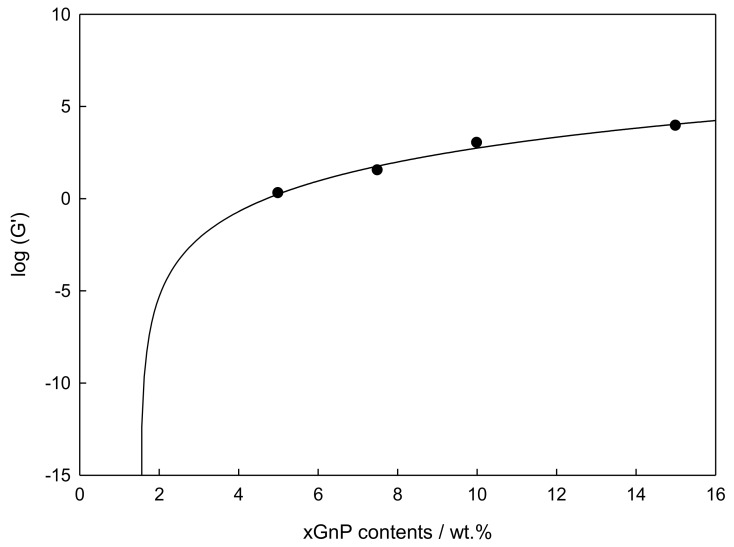
Logarithmic storage modulus, log (G′), vs. xGnP wt%, adjusted using the percolation model (Equation (2)) at 0.01rad·s^−1^ and 293.15 K with 0.1% of strain.

The IoNFs experimental low frequency equilibrium moduli were modelled as a function of concentration following a percolation equation [28,29]:

(2) $$\log(G\prime )\;=\;A\;+\;B\;\log(m-m_{0}),$$

where A and B are constant, m is the weight percentage of the graphene IoNF, m_0_ is the threshold value for m, and the equation applies only in the vicinity of the percolation transition. This correlation is shown in Figure 8.

A value of the percolation concentration of 1.5% by weight was obtained, which demonstrates the solid internal structure of the IoNF due to the graphene addition.

**Figure 9:**
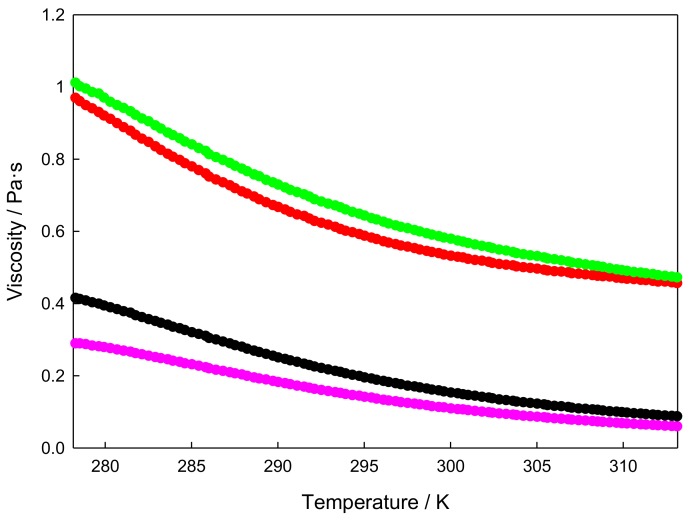
Viscosity at 10 s^−1^ shear rate versus temperature for xGnP/[C_2_C_1_py][C_4_F_9_SO_3_] at mass concentrations:(**—**) 0 wt%; (**—**) 1 wt%; (**—**) 5 wt%; and (**—**) 10 wt%.

Finally, temperature ramps were performed for the xGnP/[C_2_C_1_py][C_4_F_9_SO_3_] at the same heating rates, with a 10 s^−1^ shear rate, obtaining the sample viscosity in these conditions, as displayed in Figure 9. As expected, viscosity decreased with temperature and increased with nano additive concentration.

## 4. Conclusions

Summarizing, samples of different concentrations of IoNFs obtained by dispersing graphene nanosheets in FIL have been analyzed in this work. The characterization of these IoNFs was carried out from the evaluation of the solid–fluid transitions using a differential scanning calorimetry technique, and in addition, a viscoelastic characterization was achieved using rheology. This allowed obtaining information about the internal structure of the IoNF, determining the influence of concentration of the suspended graphene.

Previous research showed that 1-ethyl-3-ethylpyridinium perfluorobutanesulfonate is an optimum candidate to be used as a solvent in gas separation processes with a significantly reduced environmental impact. The formulation of IoNFs through the addition of graphene, or derivatives with a chemically functionalized surface, represents an option to enhance gas solubility, improving the performance in gas capture and separation.

The obtained DSC profiles indicated a very complex thermal behavior of the solid–fluid transitions for the fluorinated ionic liquid, which are typical of polymorphic systems. For the pristine base fluid, a cooling ramp showed different exothermic peaks, revealing a succession of several polymorphic crystalline phases. This trend was remarkably modified on the addition of graphene nanosheets, resulting in an extremely rich and complex phase transition behavior. The final phase transition scenario combined features of enantiotropic and polymorphic solid–solid transitions for the pure IL, and the contributions of nucleation enhancement, solid–solid transition frustration and ultimate percolated structure due to the addition of the dispersed graphene nanosheets.

The rheological profile found for these samples was also very complex. As a first and distinctive feature, all samples show non-Newtonian behavior, where modules G′ and G″ were practically constant in the low strain range, and both increased with graphene concentration, showing a clear transition from liquid-like to solid-like behavior with increasing nanoparticle load. A percolation concentration value of 1.5 wt% was obtained for this system, evidencing a solid internal structure of the IoNF.

In conclusion, the addition of graphene allowed the phase transition and viscoelastic nature of the original FIL to be tuned. Graphene concentration controlled these two patterns, and small concentration variations produced a remarkable change in the system properties. Thus, the IoNF can be precisely tailored to match the required optimal features considering the use of this system in gas separation and capture applications.

## Figures and Tables

**Table 1:** Onset temperatures and maximum peaks of xGnP/[C_2_C_1_py][C_4_F_9_SO_3_] obtained with temperature variation rates of 5 K·min^−1^.

	[C_2_C_1_py][C_4_F_9_SO_3_]	XGNP/ [C_2_C_1_py][C_4_F_9_SO_3_] 1 wt%	XGNP/ [C_2_C_1_py][C_4_F_9_SO_3_] 5 wt%	XGNP/ [C_2_C_1_py][C_4_F_9_SO_3_] 10 wt%
T_ONSET_	685 K	684 K	688 K	686 K
MAXIMUM PEAK T	710 K	713 K	705 K	712 K

**Table 2:** Experimental values of isobaric specific heat capacity of xGnP/[C_2_C_1_py][C_4_F_9_SO_3_] at the analyzed temperatures.

[C_2_C_1_py][C_4_F_9_SO_3_]		1 wt%	5 wt%	10 wt%
T (K)	*c_p_* (J·g^−1^·K^−1^)	*c_p_* (J·g^−1^·K^−1^)	*c_p_* (J·g^−1^·K^−1^)	*c_p_* (J·g^−1^·K^−1^)
293	1.296	1.321	1.359	1.401
313	1.334	1.360	1.403	1.446
333	1.367	1.391	1.442	1.482
353	1.397	1.421	1.472	1.511

**Table 3:** Crystallization, *T*_Crys_, and melting, *T*_m_, temperatures, latent heat of fusion, ∆*H*_m_, and latent heat of crystallization, ∆*H*_Crys_ of xGnP/[C_2_C_1_py][C_4_F_9_SO_3_] obtained with heating and cooling rates of 5 K·min^−1^.

Concentration, wt%	T_Crys_ (K)	T_m_ (K)	ΔH_m_ (J · g^−1^)	ΔH_Crys_(J·g^−1^)
[C_2_C_1_py][C_4_F_9_SO_3_]	250	278	38.14	34.76
1 wt% xGnP/[C_2_C_1_py][C_4_F_9_SO_3_]	268	280	41.33	40.45
5 wt% xGnP/[C_2_C_1_py][C_4_F_9_SO_3_]	258	277	41.16	39.47
10 wt% xGnP/[C_2_C_1_py][C_4_F_9_SO_3_]	255	274	40.18	39.66
